# Emergency en Bloc Resection of a Ruptured Hemangiosarcoma Anatomically Associated with the Right Retroperitoneal Space, Kidney, and Caudate Hepatic Lobe in a Dog

**DOI:** 10.3390/ani16101451

**Published:** 2026-05-09

**Authors:** Seung-Hyun Kim, Jang-Han Yoon, Chun-Sik Bae

**Affiliations:** 1 KSH Surgical Animal Medical Center, Gwangju 61617, Republic of Korea; tomcoma1@gmail.com; 2 Department of Veterinary Surgery, College of Veterinary Medicine, Chonnam National University, Gwangju 61186, Republic of Korea

**Keywords:** hemoperitoneum, hemangiosarcoma, emergency surgery, retroperitoneal mass, nephrectomy, partial hepatectomy

## Abstract

A 13-year-old, spayed female Maltese dog presented with acute collapse and profound lethargy of approximately 1 h duration. On admission, the dog was in hypovolemic shock, with severe hypotension, marked pallor, tachycardia, and hemoperitoneum identified by focused abdominal ultrasonography. Ultrasonography also revealed a ruptured hemorrhagic mass in the right retroperitoneal space. Because the patient was hemodynamically unstable and ongoing life-threatening hemorrhage was suspected, preoperative computed tomography was considered unsafe, and emergency exploratory laparotomy was performed following transfusion-based resuscitative stabilization. Intraoperatively, the lesion was found to involve the right kidney and caudate hepatic lobe. En bloc resection, including right nephrectomy, partial hepatectomy, and excision of the retroperitoneal-associated mass, was completed while preserving the right adrenal gland and adjacent major vasculature. Histopathologic and immunohistochemical evaluation supported a diagnosis of solid-pattern hemangiosarcoma, although a single primary site of origin could not be definitively assigned. The dog recovered without major perioperative complications, was discharged in stable condition, and maintained good quality of life for approximately 1 year; however, late clinical decline suspicious for recurrence or progression was not objectively confirmed. This case highlights the practical utility of focused ultrasonography, rapid surgical decision-making, and carefully planned en bloc resection for emergency hemorrhage control in a dog when advanced imaging was considered unsafe.

## Simple Summary

A 13-year-old Maltese dog presented with hemorrhagic shock after sudden collapse. Focused abdominal ultrasonography identified severe hemoperitoneum and a ruptured hemorrhagic mass in the right retroperitoneal space. Because the patient was hemodynamically unstable, computed tomography was not performed, and emergency exploratory laparotomy was undertaken immediately after resuscitative stabilization. Intraoperatively, the mass was found to involve the right kidney and the caudate hepatic lobe, and en bloc resection including right nephrectomy and partial hepatectomy was successfully completed. Histopathologic and immunohistochemical findings supported a diagnosis of solid-pattern hemangiosarcoma, although a single primary site of origin could not be definitively assigned. The dog recovered uneventfully without major perioperative complications and maintained a good quality of life for approximately 1 year after surgery before late clinical decline suspicious for recurrence or progression, which was not objectively confirmed. This case highlights the practical importance of focused ultrasonography, rapid surgical decision-making, and carefully planned en bloc resection in the emergency management of a dog with life-threatening hemoperitoneum caused by rupture of a hemorrhagic abdominal mass.

## 1. Introduction

Hemangiosarcoma (HSA) is a malignant vascular neoplasm in dogs and is clinically important because of its fragile, highly hemorrhagic nature and aggressive biologic behavior [1,2]. Although the spleen, right atrium, and liver are recognized as the most common sites of involvement, acute nontraumatic hemoperitoneum in dogs has been associated with substantial perioperative risk and frequent need for blood product support [3,4]. Retroperitoneal presentations of hemangiosarcoma appear to be uncommon and remain less well characterized than more typical visceral presentations [5,6]. In such situations, affected dogs may deteriorate rapidly because of ongoing blood loss, hypovolemia, and reduced tissue perfusion, making early recognition and timely intervention essential in emergency practice [7,8].

From a surgical standpoint, hemorrhagic masses in the right retroperitoneal region are particularly challenging because of their deep location and close association with the renal hilus, caudal vena cava, phrenicoabdominal vasculature, and adjacent organs. These lesions may therefore present as life-threatening abdominal emergencies requiring immediate hemorrhage control and technically demanding dissection in a confined operative field. When the mass encases or replaces the kidney, or extends cranially toward the caudate hepatic lobe, the complexity of surgery increases further because definitive treatment may require combined nephrectomy, hepatic resection, and meticulous retroperitoneal dissection while preserving major vascular structures [9,10,11]. Published descriptions of HSA presenting as a hemorrhagic mass in or closely associated with the retroperitoneal space, particularly with extensive renal involvement, appear to be limited, and reports detailing concurrent hepatic involvement managed by emergency en bloc resection are similarly scarce [5,11,12]. Therefore, a case-specific feature of the present report is the emergency management of a ruptured, anatomically complex hemangiosarcoma with gross retroperitoneal association and renal and caudate hepatic lobe involvement, requiring combined right nephrectomy, partial hepatectomy, and retroperitoneal dissection in a dog with hemorrhagic shock.

In dogs presenting with acute collapse and hemoperitoneum, additional diagnostic imaging and staging, including computed tomography (CT) when feasible, may improve anatomic characterization and treatment planning; however, these steps may not be practical in hemodynamically unstable patients when active hemorrhage is suspected [13,14]. In this setting, focused abdominal ultrasonography, combined with physical examination findings and immediate resuscitative assessment, can support rapid decision-making when urgent hemorrhage control is required. Several reports in dogs with nontraumatic hemoperitoneum emphasize the practical value of prompt clinical assessment and early surgical intervention when ongoing tumor-associated hemorrhage is strongly suspected [4,15,16].

The present report is therefore framed primarily as an emergency clinical and surgical case rather than as a tumor-biology-centered report. Specifically, it describes the management of a geriatric dog with acute hemorrhagic collapse in which ultrasonographic localization of a ruptured hemorrhagic mass in the right retroperitoneal space, together with the patient’s hemodynamic instability, prompted immediate transfusion support and emergency exploratory laparotomy without preoperative CT. Intraoperatively, the lesion was found to be intimately associated with the right retroperitoneal space and to involve the right kidney and caudate hepatic lobe, necessitating en bloc resection under conditions of substantial hemorrhagic and anatomic risk. Histopathologic and immunohistochemical evaluation subsequently supported a diagnosis of solid-pattern hemangiosarcoma; therefore, this report focuses on the emergency decision-making, intraoperative strategy, and perioperative management of complex abdominal surgery in an unstable patient.

Accordingly, the purpose of this report is to describe the emergency diagnostic reasoning, perioperative decision-making, and surgical management of a ruptured hemorrhagic mass anatomically associated with the right retroperitoneal space, right kidney, and caudate hepatic lobe in a dog with hemorrhagic shock. This case also illustrates the feasibility and key perioperative considerations of complex en bloc resection in a time-critical emergency setting, and may provide practical insight for surgeons managing similarly high-risk hemorrhagic abdominal masses.

## 2. Case Description

A 13-year-old, 3.5 kg, spayed female Maltese (body condition score, 5/9) presented at the emergency service with acute collapse that had developed approximately 1 h before admission. On presentation, the dog was in lateral recumbency, severely lethargic, and minimally responsive. Mucous membranes were markedly pale, capillary refill time was not measurable, and non-invasive blood pressure indicated severe hypotension (systolic arterial pressure, 70 mmHg; mean arterial pressure, 42 mmHg). Compensatory tachycardia (approximately 200 beats/min) and tachypnea were also present. Overall, the patient was considered to be in hypovolemic shock secondary to acute internal hemorrhage and was classified as American Society of Anesthesiologists (ASA) physical status V-E, indicating a moribund patient not expected to survive without the operation and requiring an emergency procedure [17].

**Figure 1:**
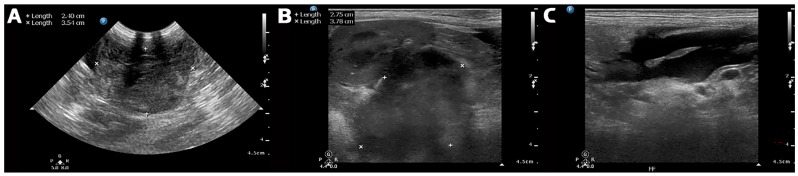
Focused abdominal ultrasonographic images of the abdominal lesion and hemoperitoneum. (**A**,**B**) Right-sided abdominal ultrasonographic views showing a hypoechoic mass located deep to the right kidney and anatomically associated with the right retroperitoneal region. (**C**) Focused abdominal ultrasonographic view showing free abdominal fluid consistent with hemoperitoneum.

Clinicopathologic evaluation revealed profound anemia (hematocrit, 16%; reference interval [RI], 37.3–61.7%; hemoglobin, 6.2 g/dL; RI, 13.1–20.5 g/dL; red blood cell count, 2.8 × 10^6^/µL; RI, 5.65–8.87 × 10^6^/µL), leukocytosis (23.3 × 10^3^/µL; RI, 5.05–16.76 × 10^3^/µL) with neutrophilia (19.83 × 10^3^/µL; RI, 2.95–11.64 × 10^3^/µL), and increased C-reactive protein (74.2 mg/L; RI, <10 mg/L). Serum biochemistry, including hepatic, renal, pancreatic, and electrolyte parameters, was within reference limits. The coagulation profile was also abnormal, with prolonged prothrombin time (17 s; RI, 11.0–15.5 s) and activated partial thromboplastin time (31 s; RI, 11.0–17.5 s), consistent with coagulation derangement in the setting of severe hemorrhage. Three-view thoracic radiographs were obtained during initial stabilization and showed no obvious evidence of pleural effusion, pulmonary metastasis, or other clinically significant thoracic abnormalities. Further abdominal staging beyond focused ultrasonography, including computed tomography, was not performed before surgery because of the patient’s severe hemodynamic instability and concern for ongoing life-threatening hemorrhage. Abdominal fluid cytology was not performed because immediate surgical hemorrhage control was prioritized. Focused abdominal ultrasonography was performed using a Philips HD15 ultrasound system (Philips Healthcare, Bothell, WA, USA) with linear and convex transducers. The examination demonstrated a large volume of free abdominal fluid consistent with hemoperitoneum and a hypoechoic, heterogeneous mass measuring approximately 4 cm in the right retroperitoneal region, deep to the right kidney (Figure 1). The lesion showed irregular margination, capsular discontinuity, adjacent echogenic fluid accumulation, and marked vascularity on Doppler assessment, raising strong suspicion of a ruptured hemorrhagic mass with recent or active hemorrhage. No other specific sonographic features were identified, and no obvious abnormalities were identified in the spleen or other major abdominal organs. Detailed Doppler settings and machine parameters were not available from the archived emergency record because the examination was performed as a focused emergency scan in a severely unstable patient. Based on these findings, rupture of a hemorrhagic mass in the right retroperitoneal region was considered the most likely cause of the acute hemoperitoneum. At this stage, the precise site of origin could not be determined, and the lesion was interpreted as a hemorrhagic mass anatomically associated with the right retroperitoneal region. Because the patient remained severely unstable and ongoing intra-abdominal hemorrhage was suspected, the decision to proceed with emergency exploratory laparotomy was made immediately after focused ultrasonographic localization of the lesion. Blood typing, cross-matching, transfusion preparation, hemodynamic support, and anesthetic preparation were performed concurrently to minimize delay, and the dog was transferred to surgery within approximately 1 h of the diagnostic assessment. The overall clinical course, including presentation, emergency decision-making, definitive surgical management, and follow-up, is summarized in Table 1.

Blood typing and major cross-matching were performed immediately, and no incompatibility was detected. Because of the patient’s hemodynamic instability and concern for ongoing life-threatening hemorrhage, preoperative CT was considered unsafe and emergency exploratory laparotomy was prioritized. Two 22-gauge intravenous catheters were placed in the cephalic veins to permit concurrent whole-blood transfusion and administration of a balanced crystalloid solution (Hartmann’s solution). Based on an estimated pre-hemorrhage hematocrit of approximately 45%, the admission hematocrit of 16%, and an estimated canine blood volume of 80–90 mL/kg, estimated blood loss was approximately 180–200 mL. The total whole-blood transfusion volume administered during the perioperative period and the first 24 h after surgery was approximately 180–200 mL, corresponding to approximately 50–60 mL/kg. Dopamine was administered as a constant-rate infusion (10 μg/kg/min, IV), titrated to effect, and intraoperative arterial blood pressure was maintained at a systolic arterial pressure of >100 mmHg and mean arterial pressure of >60 mmHg. Premedication consisted of remifentanil (1 μg/kg, IV), and after 2 min of preoxygenation, anesthesia was induced with alfaxalone (2 mg/kg, IV, to effect). Following endotracheal intubation, anesthesia was maintained with isoflurane in oxygen and a remifentanil constant-rate infusion (0.2 μg/kg/min, IV). Mechanical ventilation, including pressure-regulated volume control, was adjusted to maintain the end-tidal CO_2_ between 35 and 45 mmHg, with continuous ECG, pulse oximetry, capnography, and non-invasive blood pressure monitoring. After rapid resuscitative preparation and anesthetic stabilization, the dog was transferred directly to surgery. The total anesthesia time and skin-to-skin surgical time were approximately 1 h and less than 1 h, respectively.

**Figure 2:**
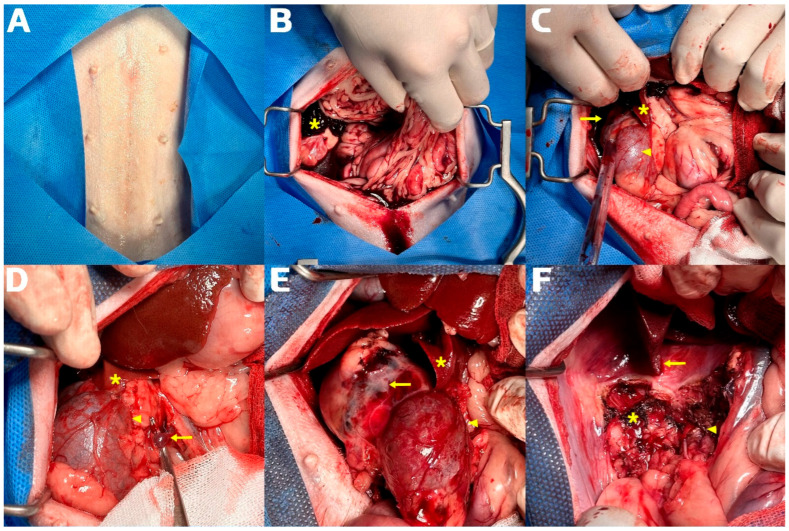
Intraoperative images of the emergency surgical procedure. (**A**) Surgical field after shaving and sterilization. (**B**) Hemoperitoneum filled with blood and large clots observed immediately after laparotomy (asterisk, large blood clot). (**C**) Identification of the bleeding mass during suction removal of blood and clots (asterisk, caudate hepatic lobe; arrowhead, right kidney; arrow, hemorrhagic mass anatomically associated with the right retroperitoneal region). (**D**) Right nephrectomy in progress (asterisk, caudate hepatic lobe; arrowhead, right kidney; arrow, right renal vein and artery just before ligation). (**E**) The mass anatomically associated with the right retroperitoneal region (asterisk, caudate hepatic lobe; arrowhead, ligated and excised right kidney; arrow, retroperitoneal-associated component of the mass). (**F**) After partial hepatectomy, right nephrectomy, and excision of the retroperitoneal-associated component of the mass (asterisk, site of excision of the retroperitoneal-associated mass component; arrowhead, nephrectomy site; arrow, partial hepatectomy site of the caudate hepatic lobe). Symbols are defined separately within each panel because the operative field differs between images and identical symbols do not necessarily indicate the same structure across panels.

A ventral midline celiotomy extending from the xiphoid to the pubis was performed to maximize exposure (Figure 2). Upon entry into the abdomen, a large volume of unclotted and partially clotted blood was encountered, confirming ongoing intra-abdominal hemorrhage. A self-retaining abdominal retractor was applied to optimize exposure, and blood and organized clots were evacuated using suction, laparotomy sponges, and atraumatic tissue handling to improve visualization of the deeper retroperitoneal structures, at which point a ruptured, highly vascular mass was identified within the right retroperitoneal region. The lesion was closely associated with the right kidney and showed active capsular bleeding from multiple sites. Temporary hemorrhage control was achieved using oxidized regenerated cellulose, direct pressure, and bipolar coagulation of focal bleeding points. Definitive dissection and hemostasis were subsequently performed using a combination of bipolar coagulation, electrocautery, sequential vessel ligation, and layered retroperitoneal dissection along identifiable tissue planes.

Further exploration revealed that the mass was grossly associated with the right renal capsule and parenchyma, with loss of recognizable normal renal architecture. The right kidney was completely engulfed, and no grossly salvageable renal tissue remained. The mass was firm, friable, and highly vascular. Cranially, the lesion was closely associated with and grossly involved the caudate hepatic lobe, necessitating concurrent hepatic resection. In contrast, the right adrenal gland remained grossly distinct from the mass and was separated by a preserved fascial plane. However, the proximity of the lesion to the phrenicoabdominal vessels, adrenal tributaries, and caudal vena cava substantially increased the risk of catastrophic hemorrhage during dissection.

Definitive resection began with isolation of the right renal pedicle. Careful blunt–sharp dissection was used to expose the renal artery, renal vein, and ureter, and vascular control was achieved with bipolar vessel sealing, electrocautery, and suture ligation. Layered retroperitoneal dissection was maintained to minimize capsular disruption and reduce the risk of avulsion or hemorrhage from short venous and perirenal vascular branches. This step required particular care because the mass distorted the normal hilar anatomy and the right renal vein drained directly into the caudal vena cava. The renal vessels were doubly ligated and transected, and the ureter was ligated and divided at a grossly uninvolved segment. Right nephrectomy was then completed as part of the planned en bloc resection.

Attention was then directed to the cranial extent of the lesion. The involved portion of the caudate hepatic lobe showed gross disruption of the parenchymal margin and direct involvement by the mass. Partial hepatectomy was performed under direct visualization along a planned transection line while avoiding major hepatic vasculature. Minor hemorrhage from the hepatic cut surface was controlled using bipolar coagulation and topical hemostatic support. The gallbladder and major portal structures were grossly unaffected.

After nephrectomy and partial hepatectomy, the portion of the mass associated with the right retroperitoneal plane was dissected free. The mass was deeply associated with the right retroperitoneal plane, displaced the caudal vena cava medially, and compressed adjacent fascial layers. Because blunt traction risked avulsion of fragile vessels or rupture of the tumor capsule, dissection proceeded in a layered and deliberate fashion with sequential sealing or ligation of small vascular branches and sharp separation along identifiable tissue planes. The major operative risks at this stage included direct caval injury, tearing of the phrenicoabdominal vasculature, retroperitoneal arterial bleeding, and uncontrolled hemorrhage associated with capsular disruption. Following prolonged and meticulous dissection, the mass, together with the completely infiltrated right kidney and the involved caudate hepatic segment, was removed en bloc. Grossly complete excision of all visible disease was achieved, and the right adrenal gland and adjacent major vasculature were preserved. Surgical margins were evaluated grossly at the time of surgery; histologic margin assessment was not performed because the lesion was removed as a large, anatomically complex en bloc specimen without orientation for formal margin mapping.

The abdomen was extensively lavaged with warm sterile saline, and residual blood clots and debris were removed. A systematic exploratory assessment of the remaining liver, spleen, mesentery, gastrointestinal tract, diaphragm, and residual retroperitoneum did not reveal additional gross lesions, active bleeding sites, or visible metastatic nodules. After confirmation of hemostasis, the abdomen was closed routinely.

Postoperatively, the dog was hospitalized in the intensive care unit for 7 days and received continued whole-blood transfusion support for approximately 24 h, balanced crystalloid therapy, opioid analgesia, antimicrobial treatment, and close hemodynamic monitoring. Analgesia was provided with hydromorphone (0.02 mg/kg/h IV constant-rate infusion) for approximately 2 days after surgery, after which the patient was transitioned to oral codeine-based analgesia during the postoperative recovery period. Antimicrobial therapy consisted of piperacillin/tazobactam (50 mg/kg IV q6h) and marbofloxacin (2 mg/kg IV q24h). Serial abdominal ultrasonography and repeated hematologic evaluations during hospitalization showed no evidence of recurrent intra-abdominal bleeding, and the hematocrit increased to 45% after a total whole-blood transfusion volume of approximately 180–200 mL during the perioperative period and the first 24 h after surgery. Despite right nephrectomy and partial hepatectomy, renal variables remained acceptable (blood urea nitrogen, 36 mg/dL; creatinine, 1.5 mg/dL). Alkaline phosphatase and alanine aminotransferase were increased (398 and 376 U/L), but total bilirubin remained acceptable (0.3 mg/dL), with no evidence of hepatic insufficiency. The patient’s attitude improved by postoperative day 2, spontaneous appetite returned by day 3, and no major complications developed during hospitalization. The dog was discharged in stable condition on postoperative day 7. Follow-up examinations were performed at approximately 3-month intervals and included abdominal ultrasonography, complete blood count (CBC), and serum biochemistry. Postoperative advanced staging, including thoracic and abdominal CT, was recommended on multiple occasions; however, it was not performed because the owner declined further staging and oncologic treatment for practical reasons. During these periodic recheck examinations, the dog maintained good appetite, activity, and overall quality of life, and no clinically significant abnormalities prompting additional intervention were identified. No adjuvant chemotherapy was administered because the owner declined further treatment. Approximately 1 year after surgery, late clinical decline developed and was considered clinically suspicious for recurrence or progression; however, objective confirmation was not obtained.

**Figure 3:**
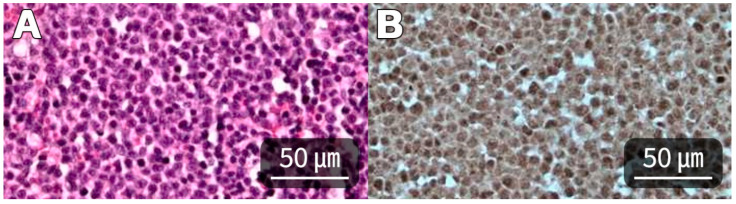
Histopathological and immunohistochemical features of the excised mass. (**A**) The neoplasm is composed predominantly of infiltrative solid sheets and interlacing cords of atypical endothelial cells with scant to absent overt vascular channel formation. Focal vasoformative areas and multifocal hemorrhage are evident. Hematoxylin and eosin stain; original magnification ×400; scale bar = 50 μm. (**B**) The neoplastic cells exhibit positive immunoreactivity for von Willebrand factor (vWF), supporting endothelial differentiation. Immunohistochemistry; original magnification ×400; scale bar = 50 μm. These findings supported a diagnosis of solid-pattern hemangiosarcoma but did not establish a definitive primary site of origin.

Histopathologic and immunohistochemical assessment of the resected mass was performed by a commercial reference laboratory. The tumor was composed predominantly of infiltrative sheets and interlacing cords of atypical endothelial cells with scant to absent overt vascular channel formation, consistent with a solid-pattern growth pattern. Focal vasoformative areas and marked multifocal hemorrhage were also present. Immunolabeling for vimentin and von Willebrand factor supported endothelial differentiation, and the overall findings were consistent with solid-pattern hemangiosarcoma (Figure 3). The association with the right retroperitoneal space and the concurrent renal and hepatic involvement were determined from imaging and intraoperative findings rather than histopathology alone. Accordingly, the lesion was interpreted conservatively as a ruptured hemangiosarcoma closely associated with the right retroperitoneal space and involving the right kidney and caudate hepatic lobe, without definitive assignment of a single site of origin. Written informed owner consent was obtained for treatment and publication of anonymized clinical information and images. Ethical review was waived because this case report describes routine clinical care in a client-owned dog without experimental intervention.

## 3. Discussion

This case highlights the practical importance of time-critical decision-making in a dog with acute nontraumatic hemoperitoneum and hemodynamic instability. Although HSA is a well-recognized malignant vascular neoplasm in dogs, most clinical attention has focused on splenic, hepatic, and right atrial presentations, whereas hemorrhagic masses associated with the retroperitoneal space appear to be described less commonly [1,2,3,6]. In the present dog, the clinically decisive issue at admission was not the histologic identity of the lesion, but the combination of acute collapse, profound anemia, severe hypotension, ultrasonographic evidence of marked hemoperitoneum, and suspicion of ongoing active hemorrhage. Under these circumstances, the need for immediate hemorrhage control outweighed the potential benefit of additional preoperative imaging. This distinction is relevant to emergency decision-making because, in unstable dogs with suspected tumor-associated hemorrhage, immediate hemorrhage control may take precedence over further diagnostic characterization when additional delay is judged to increase clinical risk [4,7,8]. In this context, the present case is notable for the combination of life-threatening hemoperitoneum, gross retroperitoneal association, complete right renal involvement, caudate hepatic lobe involvement, and the need for emergency multiorgan en bloc resection without preoperative CT staging.

In the present case, focused ultrasonography, when interpreted together with physical examination and hemodynamic assessment, provided sufficient clinical information to proceed with emergency exploratory laparotomy. CT remains valuable for anatomic mapping and operative planning, particularly for deep retroperitoneal lesions, but the dog’s profound shock and suspected ongoing hemorrhage made immediate operative hemorrhage control the clinical priority in this case [13,14]. Consequently, the absence of preoperative CT limited complete staging and precise preoperative assessment of the lesion’s full anatomic extent. This limitation also reduced confidence in assigning a primary site of origin and restricts the reproducibility of the surgical approach, because operative planning relied substantially on focused ultrasonography and intraoperative findings. Therefore, the operative strategy described here should be interpreted as a case-specific emergency approach based on intraoperative findings, rather than as a standardized protocol that can be broadly generalized to all similar hemorrhagic retroperitoneal-associated masses. The lack of complete staging also limits oncologic interpretation, because occult metastatic disease or residual disease could not be definitively excluded. In this dog, prompt ultrasonographic localization and immediate surgical exploration allowed timely hemorrhage control and survival to discharge. This observation supports the practical relevance of rapid decision-making in a severely unstable patient, while recognizing that complete preoperative anatomic staging remains valuable whenever the patient’s condition permits [4,15,16].

From a surgical perspective, this case was notable because the mass was closely associated with the right retroperitoneal space and had grossly involved both the right kidney and the caudate hepatic lobe, creating a combination of oncologic and hemostatic challenges. Retroperitoneal surgery is inherently demanding in small dogs because of limited operative working space and the close proximity of the renal hilus, caudal vena cava, phrenicoabdominal vessels, adrenal tributaries, and surrounding fascial planes [9,10]. In this case, definitive treatment required not only removal of a bleeding mass, but also safe dissection around major vascular structures while controlling ongoing hemorrhage and preserving uninvolved adjacent tissue, including the right adrenal gland. The lesion’s replacement of normal renal architecture precluded organ-sparing management, and gross involvement of the caudate hepatic lobe necessitated partial hepatectomy as part of the same en bloc procedure. Published descriptions of comparable cases appear limited, particularly when extensive renal involvement and concurrent caudate hepatic lobe involvement are encountered in the setting of emergency surgery [5,11,12]. Compared with previously reported retroperitoneal or renal hemangiosarcomas, the present case adds a detailed description of emergency decision-making and operative sequencing for a multiorgan-associated hemorrhagic mass requiring combined nephrectomy, partial hepatectomy, and retroperitoneal dissection.

From a technical standpoint, this case is most relevant for the operative strategy used to achieve hemorrhage control and gross removal of visible disease. The procedure required staged management of the hemorrhagic abdomen, identification of the main bleeding lesion, systematic hilar control, controlled hepatic transection, and layered retroperitoneal dissection performed under conditions of substantial vascular risk. In similar cases, the greatest intraoperative danger may arise not simply from tumor removal itself, but from distortion of normal anatomy by hemorrhage, friable neoplastic tissue, and close adherence to short venous structures. From a practical surgical standpoint, key factors that likely contributed to the favorable perioperative outcome in this case were rapid evacuation of hemoperitoneum to restore anatomic orientation, early temporary hemorrhage control, deliberate hilar isolation before traction, and layered retroperitoneal dissection to avoid avulsion of short venous branches and uncontrolled bleeding. This case illustrates that, even in a geriatric small-breed dog with severe preoperative instability, complex en bloc resection may be achievable when the operative plan prioritizes immediate hemostasis, sequential vascular control, and careful preservation of major adjacent structures. In that sense, the present report contributes most directly to emergency abdominal surgery rather than to comparative tumor pathology.

Histopathologic and immunohistochemical examination subsequently supported a diagnosis of solid-pattern hemangiosarcoma. This finding is clinically relevant because solid-pattern or poorly vasoformative HSA may be less visually intuitive than classic blood-filled vasoformative lesions, particularly when the surgeon is confronted primarily with a ruptured hemorrhagic mass rather than a well-demarcated organ-based tumor [1,2]. However, in the present case, histopathology served principally to define the tumor type after surgery, whereas the anatomic association with the right retroperitoneal space, right kidney, and caudate hepatic lobe was interpreted from ultrasonographic localization and direct intraoperative assessment. This distinction is clinically relevant because the exact anatomic origin of deeply seated hemorrhagic abdominal masses may not always be assignable from histopathology alone. Several interpretations are possible, including a primary renal hemangiosarcoma with retroperitoneal and hepatic involvement, a primary retroperitoneal hemangiosarcoma secondarily involving the kidney and caudate hepatic lobe, or a primary hepatic hemangiosarcoma involving adjacent structures. Because histopathology did not identify a single point of origin and preoperative CT staging was not available, these alternatives could not be distinguished with confidence. Therefore, the lesion is best described conservatively as an anatomically complex, multiorgan-associated hemangiosarcoma with gross association with the right retroperitoneal space and involvement of the right kidney and caudate hepatic lobe, rather than as a tumor with a definitively assigned single primary origin.

From an oncologic perspective, the present outcome should be interpreted in the context of the generally guarded prognosis of canine visceral and retroperitoneal hemangiosarcoma. In dogs with surgically resected retroperitoneal hemangiosarcoma, median progression-free survival and overall survival have been reported as 77.5 and 168 days, respectively; median overall survival was 129.5 days after surgery alone and 241.5 days after surgery followed by adjuvant doxorubicin [6]. In renal hemangiosarcoma, nephrectomy was associated with a median survival time of 278 days overall, but dogs with hemoperitoneum had markedly shorter survival than those without hemoperitoneum, 62 versus 286 days [12]. Although caudate hepatic lobe involvement was present in this dog, a primary hepatic origin was not confirmed; therefore, comparison with primary hepatic hemangiosarcoma should be made cautiously. More broadly, visceral hemangiosarcoma is associated with aggressive biologic behavior, and surgery alone is generally considered palliative, whereas doxorubicin-based chemotherapy has been associated with longer survival in several reports [2]. Accordingly, the approximately 1-year period of acceptable postoperative function in this case should be interpreted as an individual clinical course after emergency hemorrhage control, not as evidence that surgery alone achieved unexpected oncologic control.

The postoperative course further illustrates the potential clinical relevance of decisive surgical management in this selected emergency patient. Despite severe preoperative shock and the extent of resection required, the dog recovered without major perioperative complications, showed no evidence of recurrent intra-abdominal bleeding during hospitalization on serial abdominal ultrasonography and hematologic reassessment, and maintained acceptable renal and hepatic biochemical parameters after right nephrectomy and partial hepatectomy. The patient was discharged in stable condition on postoperative day 7. Follow-up was conducted at approximately 3-month intervals and included abdominal ultrasonography, complete blood count, and serum biochemistry, during which the dog maintained good appetite, activity, and overall quality of life for approximately 1 year, although recurrence-free or metastasis-free status could not be objectively confirmed. No adjuvant chemotherapy was administered because the owner declined additional oncologic treatment. Late clinical decline subsequently developed and was considered suspicious for recurrence or progression, although objective confirmation was not obtained. Although this single-case outcome cannot be generalized, it suggests that emergency definitive surgery was associated with survival to discharge and acceptable short- to medium-term postoperative function in this individual patient, despite the anatomic complexity of the lesion. Importantly, this case should not be interpreted as evidence that surgery alone reliably improves long-term oncologic outcome in anatomically complex deep abdominal HSA. Instead, it documents a clinical scenario in which timely operative intervention enabled hemorrhage control, stabilization, and a period of acceptable postoperative function in one critically unstable dog.

Several limitations should be acknowledged. First, this is a single-case report, and no broader conclusions regarding prognosis, oncologic behavior, or treatment recommendations can be drawn. Second, preoperative CT and additional abdominal staging beyond focused ultrasonography were not performed because of hemodynamic instability, which limited preoperative anatomic characterization, formal surgical planning, and complete staging; abdominal fluid cytology was also not performed because immediate surgical hemorrhage control was prioritized. Third, the precise anatomic origin of the lesion could not be established by histopathology alone and was instead inferred from ultrasonographic localization and intraoperative findings. Fourth, although long-term follow-up was performed at approximately 3-month intervals and included abdominal ultrasonography, CBC, and serum biochemistry, postoperative advanced staging with thoracic and abdominal CT was declined by the owner. Therefore, complete removal of the mass and the presence or absence of metastatic disease could not be objectively verified by postoperative cross-sectional imaging, and late clinical decline suspicious for recurrence or progression was not objectively confirmed. Finally, adjuvant oncologic treatment was not administered because the owner declined further therapy, which further limits interpretation of long-term oncologic outcome. These limitations substantially restrict interpretation of tumor origin, staging completeness, long-term oncologic outcome, and the potential benefit of surgery alone. Nevertheless, the case remains clinically relevant as a description of high-acuity emergency surgical management, in which focused ultrasonography, rapid hemodynamic assessment, and carefully executed en bloc resection were followed by definitive hemorrhage control, survival to discharge, and approximately 1 year of acceptable postoperative function and quality of life.

## 4. Conclusions

This case describes an unstable dog with catastrophic hemoperitoneum caused by rupture of a deep abdominal hemorrhagic mass, in which focused abdominal ultrasonography, rapid hemodynamic assessment, and prompt exploratory laparotomy enabled definitive hemorrhage control and successful en bloc resection despite the absence of preoperative CT. Despite limited staging, lack of adjuvant chemotherapy, and lack of objective confirmation of late recurrence or progression, the patient survived for approximately 1 year with acceptable postoperative function and quality of life. Although this approach should not be generalized to all dogs with deep abdominal hemorrhagic masses, this descriptive case may provide case-specific practical insight into emergency surgical decision-making and perioperative management for a carefully selected unstable patient.

## Figures and Tables

**Table 1:** Clinical timeline of presentation, emergency decision-making, surgery, and follow-up.

Time Point	Clinical Events and Management
1 h before admission	Acute collapse at home.
Day 0, admission	Severe lethargy, pallor, hypotension, tachycardia, and tachypnea consistent with hypovolemic shock.
Day 0, initial diagnostics	Profound anemia and inflammatory leukogram; serum biochemistry within reference limits. Focused abdominal ultrasonography identified marked hemoperitoneum and an approximately 4 cm right retroperitoneal-associated mass suspicious for active hemorrhage.
Day 0, emergency decision-making	Because of hemodynamic instability and suspected ongoing life-threatening hemorrhage, preoperative CT and further abdominal staging were not performed, and emergency exploratory laparotomy was prioritized.
Day 0, stabilization and anesthesia	Blood typing, cross-matching, whole-blood transfusion, crystalloid therapy, dopamine-assisted hemodynamic support, and anesthetic preparation were initiated.
Day 0, surgery	Emergency en bloc resection was performed, including right nephrectomy, partial caudate hepatectomy, and excision of the retroperitoneal-associated mass component, while preserving the right adrenal gland and adjacent major vasculature.
Postoperative days 2–3	Attitude improved by day 2, and spontaneous appetite returned by day 3.
Postoperative day 7	Discharged in stable condition without major perioperative complications.
Postoperative histopathology and immunohistochemistry	Findings supported solid-pattern hemangiosarcoma; anatomic association with the right retroperitoneal space and renal/hepatic involvement was interpreted from imaging and intraoperative findings rather than histopathology alone.
Periodic recheck examinations	Follow-up at approximately 3-month intervals included abdominal ultrasonography, complete blood count, and serum biochemistry. No adjuvant chemotherapy was administered because the owner declined further treatment.
Approximately 1 year after surgery	Late clinical decline suspicious for recurrence or progression; objective confirmation was not obtained.

## Data Availability

All data are contained within the article.
